# Pregnancy outcomes in women with a mitral valve prosthesis: A systematic review and meta-analysis

**DOI:** 10.1016/j.xjon.2023.05.001

**Published:** 2023-05-09

**Authors:** Pepijn Grashuis, Shanti D.M. Khargi, Kevin Veen, Azzeddine el Osrouti, Shirin Bemelmans-Lalezari, Jérôme M.J. Cornette, Jolien W. Roos-Hesselink, Johanna J.M. Takkenberg, Mostafa M. Mokhles

**Affiliations:** a Department of Cardiothoracic Surgery, Erasmus Medical Centre, Rotterdam, The Netherlands; b Department of Cardiothoracic Surgery, University Medical Centre Utrecht, Utrecht, The Netherlands; c Department of Obstetrics and Fetal Medicine, Erasmus Medical Centre, Rotterdam, The Netherlands; d Department of Cardiology, Erasmus Medical Centre, Rotterdam, The Netherlands

**Keywords:** mitral valve replacement, pregnancy, bioprosthesis, mechanical valve, oral anticoagulant

## Abstract

**Objectives:**

To evaluate the ongoing debate concerning the choice of valve prosthesis for women requiring mitral valve replacement (MVR) and who wish to conceive. Bioprostheses are associated with risk of early structural valve deterioration. Mechanical prostheses require lifelong anticoagulation and carry maternal and fetal risks. Also, the optimal anticoagulation regimen during pregnancy after MVR remains unclear.

**Methods:**

A systematic review and meta-analysis was conducted of studies reporting on pregnancy after MVR. Valve- and anticoagulation-related maternal and fetal risks during pregnancy and 30 days’ postpartum were analyzed.

**Results:**

Fifteen studies reporting 722 pregnancies were included. In total, 87.2% of pregnant women had a mechanical prosthesis and 12.5% a bioprosthesis. Maternal mortality risk was 1.33% (95% confidence interval [CI], 0.69-2.56), any hemorrhage risk 6.90% (95% CI, 3.70-12.88). Valve thrombosis risk was 4.71% (95% CI, 3.06-7.26) in patients with mechanical prostheses. 3.23% (95% CI, 1.34-7.75) of the patients with bioprostheses experienced early structural valve deterioration. Of these, the mortality was 40%. Pregnancy loss risk was 29.29% (95% CI, 19.74-43.47) with mechanical prostheses versus 13.50% (95% CI, 4.31-42.30) for bioprostheses. Switching to heparin during the first trimester demonstrated a bleeding risk of 7.78% (95% CI, 3.71-16.31) versus 4.08% (95% CI, 1.17-14.28) for women on oral anticoagulants throughout pregnancy and a valve thrombosis risk of 6.99% (95% CI, 2.08-23.51) versus 2.89% (95% CI, 1.40-5.94). Administration of anticoagulant dosages greater than 5 mg resulted in a risk of fetal adverse events of 74.24% (95% CI, 56.11-98.23) versus 8.85% (95% CI, 2.70-28.99) in ≤5 mg.

**Conclusions:**

A bioprosthesis seems the best option for women of childbearing age who are interested in future pregnancy after MVR. If mechanical valve replacement is preferred, the favorable anticoagulation regimen is continuous low-dose oral anticoagulants. Shared decision-making remains priority when choosing a prosthetic valve for young women.

## Video Abstract

**Graphical abstract:**

**Figure:**
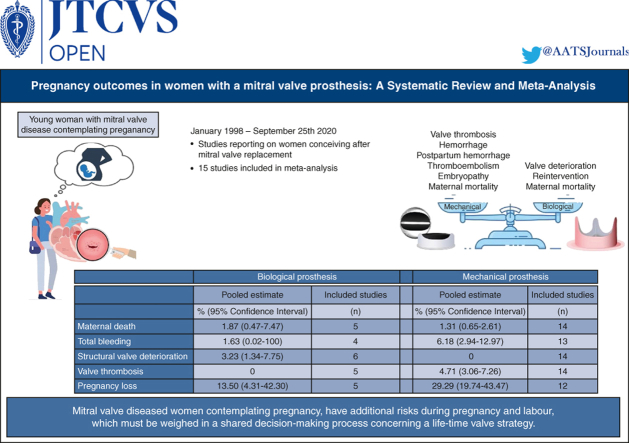

**Figure:**
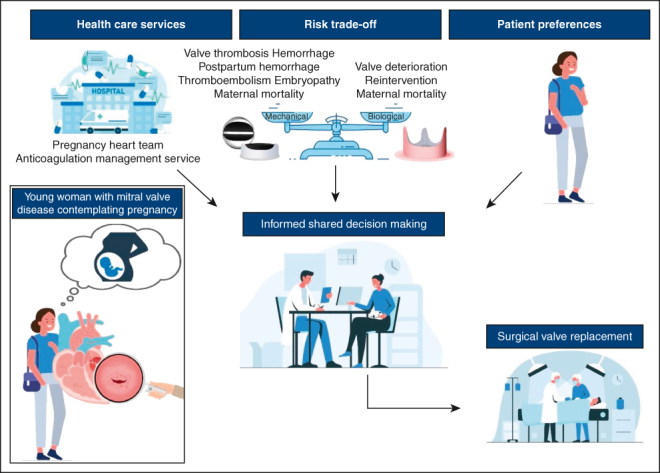
Shared decision-making in women with mitral valve disease who contemplate pregnancy.

**Central Message**

Pregnancy with a mitral valve prosthesis is associated with risks for adverse events during pregnancy and 30 days’ postpartum. These must be weighed in a preoperative shared decision-making process.

**Perspective**

An ideal heart valve prosthesis for women who contemplate pregnancy does not exist. Bioprostheses are subject to valve deterioration, leading to possible risks of early reintervention. Mechanical valve implantation and the accompanied anticoagulation regimen could increase pregnancy-related risks of thrombotic and hemorrhagic events and embryopathy.

Women of childbearing age with severe mitral valve disease require mitral valve replacement (MVR) before pregnancy if a repair of the native valve is not feasible. If left unreplaced before pregnancy, the physiological increase in cardiac output during pregnancy could lead to cardiac decompensation.^1^ MVR can be performed through the implantation of a bioprosthetic or a mechanical valve prosthesis. The wish to conceive after MVR could influence the preoperative decision in favor of the valve that facilitates safe and optimal maternal and pregnancy outcomes. However, evidence on the optimal valve prosthesis for this specific population is scarce, which is concerning, as implantation of either a bioprosthetic or mechanical valve prosthesis is associated with maternal and fetal risks both during and after pregnancy.^2^

A biological prosthesis is known for its limited durability, and implantation in young patients may be associated with accelerated structural valve deterioration (SVD) and consecutive reoperation.^3^ Alternatively, a mechanical valve is designed to last a lifetime and is not subject to deterioration. However, a mechanical valve is thrombogenic, requiring a lifetime commitment to anticoagulation therapy to prevent adverse thromboembolic events at an increased risk of bleeding.^4^

The management of anticoagulation during pregnancy in women with a mechanical mitral valve prosthesis remains a challenging balance, since pregnancy is paired with a maternal hypercoagulable and delivery is associated with increased risks of obstetric hemorrhage.^5^ A consensus on the safest anticoagulation regimen for both mother and fetus has not yet been reached. Common oral anticoagulants (OACs) cross the placenta and can be teratogenic and induce fetal anticoagulation.^6^ Alternatively, it is possible to consider a temporary switch to the nonteratogenic heparin during both the first trimester and peripartum, although this requires subcutaneous injections, on-time pregnancy awareness, and is associated with increased rates of maternal valve-related complications such as valve thrombosis.^7^

To help inform on the choice of valve prosthesis for young women requiring MVR and a wish for future pregnancy, this systematic review aims to provide an overview of the available evidence on maternal and fetal outcomes of women who conceived after surgical MVR with either a biological or mechanical prosthesis. Our analysis also addresses the safety of the practiced anticoagulation strategies during pregnancy in women with mechanical mitral valves (see Video Abstract).

## Patients and Methods

### Protocol and Inclusion Criteria

This study followed the Preferred Reporting Items for Systematic Review and Meta-Analysis guidelines and was approved by the Erasmus MC Medical Ethics Review Board (MEC-2015-170, March 23, 2015).^8^ Studies were considered eligible if the study population consisted of women who conceived after MVR. Studies published after January 1, 1998, including 10 or more pregnancies after MVR, and reporting at least 1 of our outcomes of interest (Table E1) were included. Exclusion criteria are presented in the Appendix E1.

### Search and Study Selection

On September 25, 2020, a literature search was conducted in Embase, Medline Ovid, Web of Science, and the Cochrane Library by a biomedical information specialist in consultation with the authors. The search terms and study selection process are described in the Appendix E1.

### Subgroup Analysis

We conducted 3 subgroup analyses. Subgroups were based on the type of valve prosthesis (ie, mechanical prosthesis, biological prosthesis) and the anticoagulation regimen followed during pregnancy.

For the latter subgroup, the studies were screened for coherence with the anticoagulation regimens described in the 2018 European Society of Cardiology guidelines for management of cardiovascular disease during pregnancy.^9^ Subsequently, the studies were selected for 1 of the following subgroups: group A included OACs such as warfarin, acenocoumarol, and phenprocoumon throughout pregnancy and a switch to heparin 2 to 7 days before the expected delivery date or planned cesarean delivery. Group B included patients on OACs at conception, a switch to heparin during the sixth week up to the 12th week, followed by a switch back to OACs for the second and third trimester, and another switch back to heparin 2 to 7 days before the expected delivery date or planned cesarean delivery. The use of either unfractionated heparin (UFH) or low-molecular weight heparin (LMWH) was eligible for inclusion in group B. An overview of the anticoagulation regimens is presented in Table E2. A third subgroup analysis was performed within group A to explore differences in maternal and pregnancy outcomes with patients who took a high (>5 mg daily) versus a low dose (≤5 mg daily) of warfarin throughout pregnancy.

### Data Extraction

The reviewers extracted data independently from the studies using a data collection form in Microsoft Excel (Microsoft Corporation). The data-extraction process and information about definitions are described in the Appendix E1. Tables E3 and E4 in the Appendix E1 present the extracted maternal and pregnancy outcomes for each individual study.

### Statistical Analysis

Continuous variables are presented as mean ± standard deviation. Discrete variables are presented as proportions. Baseline characteristics are analyzed and described as a proportion of the total number of patients. All other outcome measures are described as a proportion of the total number of pregnancies. A random-effects meta-analysis was performed using R and Rstudio, loaded with the ‘metafor’ package. The pooling method is described in the Methods in the Appendix E1.

Heterogeneity is explored by subgroup analysis. However, not all baseline characteristics facilitate the creation of subgroups across the included studies. In meta-regression analysis, we explored other causes of heterogeneity.

### Quality Assessment and Sensitivity Analysis

The methodologic quality of each included study was assessed according to the Newcastle–Ottawa Scale. The influence of potential publication bias on pooled risks was investigated by sensitivity analyses by temporarily excluding the smallest quartile of studies. To assess the robustness of the results and the influence of the increasing quality of health care, an additional sensitivity analysis was performed, including studies that were published after 2010. More information about the quality assessment and sensitivity analyses is described in the Appendix E1.

## Results

### Search Results

**Figure 1:**
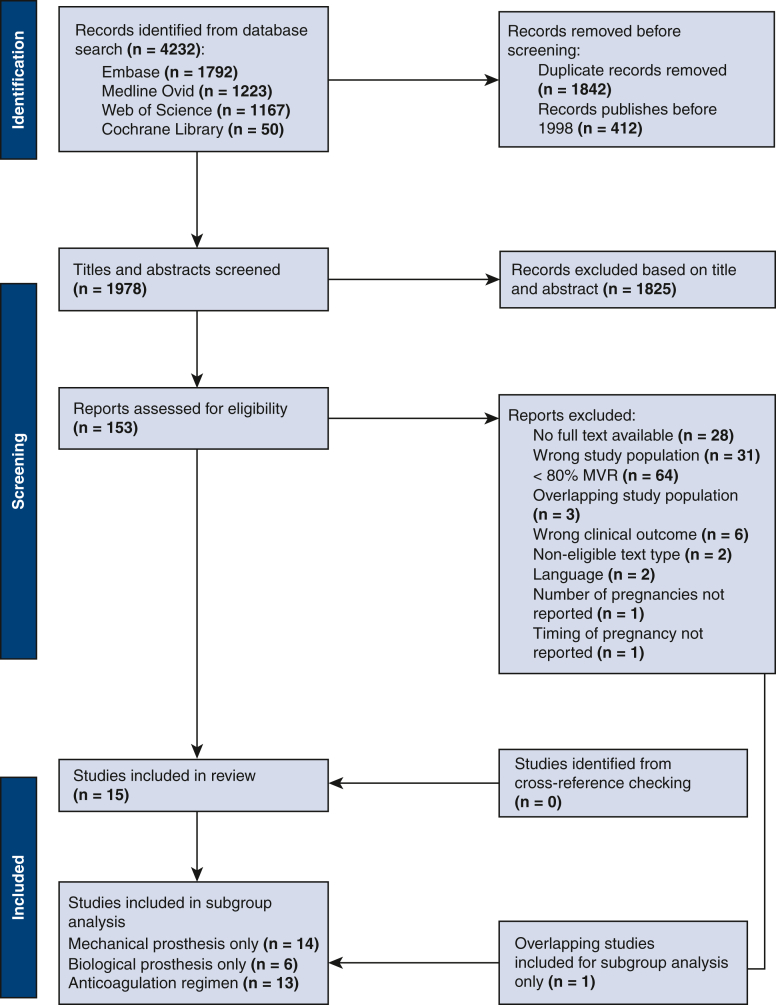
Flowchart of reference search, screening, and selection procedure. *MVR*, Mitral valve replacement.

An overview of the literature selection process is presented in Figure 1. In total, 16 studies were considered eligible for this meta-analysis,^10^, ^11^, ^12^, ^13^, ^14^, ^15^, ^16^, ^17^, ^18^, ^19^, ^20^, ^21^, ^22^, ^23^, ^24^ of which 1 study was included for subgroup analysis only because of overlapping population.^25^ The individual study characteristics are listed in the Appendix E1, Table E5.

### Baseline Characteristics

The 15 included studies encompassed a total of 722 pregnancies in 597 women with 632 valve prostheses, of which 91.0% were implanted in the mitral position. Most of the valve prostheses were mechanical valves (87.2% vs 12.5% bioprostheses). Pooled maternal mean age at pregnancy was 27.99 (±3.14) years (Table 1).

**Table 1:** Study and patient baseline characteristics of overall meta-analysis study selection

Characteristics	Overall group	Mechanical prosthesis	Biological prosthesis
Number of studies	15	14	6
Median year of patient inclusion (IQR)	2002 (1989-2007)	2004 (1994-2007)	1989 (1988-2002)
Number of patients	597	514	120
Number of pregnancies	722	613	155
Mean age at pregnancy, y ± SD	27.99 ± 3.14	29.10 ± 4.34	27.26 ± 4.66
Atrial fibrillation, %	36.80%	42.20%	36.76%
Mitral valve prostheses, n (%)	575 (90.98)	497 (92.04)	114 (93.44)
Prostheses in nonmitral position, n (%)	57 (9.02)	43 (7.96)	8 (6.56)

*IQR*, Interquartile range, *SD*, standard deviation.

### Meta-Analysis

Pooled risks of maternal and pregnancy outcomes during pregnancy or within 30 days’ postpartum are presented in Table 2.

**Table 2:** Maternal and pregnancy risks during pregnancy and within 30 d postpartum of overall meta-analysis study selection

Study outcomes	Overall (n = 15)		
	Pooled risk	Heterogeneity I^2^, %	Included studies, n
Maternal outcomes			
Maternal death, %	1.33 (95% CI, 0.69-2.56)	0%	14
Cardiac reintervention, %	2.60 (95% CI, 1.28-5.29)	3%	8
Any bleeding, %	6.90 (95% CI, 3.70-12.88)	77%	13
Obstetric bleeding, %	4.76 (95% CI, 2.64-8.59)	55%	12
Thromboembolism, %	0.94 (95% CI, 0.22-3.96)	67%	13
Valve thrombosis, %	4.01 (95% CI, 2.55-6.31)	18%	14
Stroke, %	1.54 (95% CI, 0.46-5.14)	39%	6
Heart failure, %	3.19 (95% CI, 1.03-9.83)	58%	6
Pregnancy outcome			
Pregnancy loss, %	29.08 (95% CI, 20.94-40.41)	82%	14
Stillbirth, %	3.49 (95% CI, 1.79-6.83)	64%	14
Miscarriage, %	15.36 (95% CI, 9.92-23.77)	80%	14
Termination of pregnancy, %	3.70 (95% CI, 1.26-10.91)	90%	14
Fetal loss due to maternal death, %	0.52 (95% CI, 0.10-2.73)	13%	14
Delivered alive, %	65.89 (95% CI, 57.85-75.04)	46%	14
Congenital malformation, %	1.89 (95% CI, 0.85-4.19)	32%	13
Anticoagulant embryopathy, %	0.40 (95% CI, 0.03-5.41)	78%	11
Total neonatal mortality, %	1.40 (95% CI, 0.59-3.32)	5%	14
Preterm birth, %	5.56 (95% CI, 3.57-8.67)	26%	12
SGA, %	5.13 (95% CI, 1.69-15.55)	77%	7

*CI*, Confidence interval; *SGA*, small for gestational age.

### Subgroup Analysis

#### Prosthesis type

Six studies reported on pregnant women with bioprosthetic mitral valves, encompassing a total of 155 pregnancies.^10^, ^11^, ^12^^,^^17^^,^^20^^,^^25^ In total, 23 women (14.8%) received prophylactic anticoagulant therapy due to chronic atrial fibrillation. A total of 14 studies reported on patients with mechanical prostheses, encompassing a total of 613 pregnancies.^11^, ^12^, ^13^, ^14^, ^15^, ^16^, ^17^, ^18^, ^19^, ^20^, ^21^, ^22^, ^23^, ^24^ Maternal and pregnancy outcomes of both groups are presented in Table 3.

**Table 3:** Subgroup analysis for pregnancy outcomes after implantation with biological versus mechanical valve prostheses

	Biological prosthesis			Mechanical prosthesis		
	Pooled risk	Heterogeneity I^2^, n	Included studies, n	Pooled risk	Heterogeneity I^2^, n	Included studies, n
Maternal death, %	1.87 (95% CI, 0.47-7.47)	0%	5	1.31 (95% CI, 0.65-2.61)	0%	14
Total bleeding, %	1.63 (95% CI, 0.02-100)	72%	4	6.18 (95% CI, 2.94-12.97)	81%	13
Obstetric bleeding, %	1.63 (95% CI, 0.02-100)	72%	4	4.86 (95% CI, 2.66-8.88)	53%	12
Structural valve deterioration, %	3.23 (95% CI, 1.34-7.75)	0%	6	0	–	14
Valve thrombosis, %	0	–	5	4.71 (95% CI, 3.06-7.26)	15%	14
Pregnancy loss, %	13.50 (95% CI, 4.31-42.30)	33%	5	29.29 (95% CI, 19.74-43.47)	85%	12
Neonatal mortality, %	2.97 (95% CI, 0.96-9.21)	0%	4	0.70 (95% CI, 0.15-3.29)	24%	12

*CI*, Confidence interval.

Maternal mortality occurred in 1.87% (95% confidence interval [CI], 0.47-7.47) of pregnancies with a biological prosthesis. There were 2 deaths reported of women who both experienced acute bioprosthetic valve failure during pregnancy 4.3 and 4.75 years after implantation. Both patients originated from a developing country. In women with mechanical valves, maternal death occurred in 1.31% (95% CI, 0.65-2.61) of the pregnancies.

After biological valve implantation, SVD during pregnancy or within 30 days’ postpartum occurred in 3.23% (95% CI, 1.34-7.75) of the pregnancies. Two of five (40%) women in whom SVD occurred died shortly after presentation with SVD during pregnancy. Another woman with bioprosthetic SVD was reoperated. She received a mechanical valve prosthesis instead. Two studies reported long-term follow-up of patients who had bioprostheses in situ and conceived afterwards.^10^^,^^25^ These 2 studies reported a pooled linearized SVD occurrence rate of 6.22%/patient-year (95% CI, 2.07-18.74) with a freedom from SVD at 8 years after implantation of 67.69%. In contrast, 4.71% (95% CI, 3.06-7.26) of the patients with a mechanical prosthesis developed valve thrombosis during pregnancy or within 30 days’ postpartum. Two patients (13.3%) died of 15 who experienced valve thrombosis and for whom the survival status and therapy after valve thrombosis is known. In 11 (73.3%) of these patients, the thrombosed valve was surgically replaced. For the other 2 patients, thrombolytic therapy seemed successful.

### Anticoagulant Drug Therapy

Ten studies reported outcomes of 375 pregnancies in patients who were treated according to regimen A (group A).^11^, ^12^, ^13^^,^^15^, ^16^, ^17^, ^18^^,^^20^^,^^22^^,^^24^ Treatment according to regimen B was reported in 5 studies for 100 pregnancies (Group B).^11^^,^^14^^,^^18^^,^^19^^,^^23^ Pooled proportions of maternal- and pregnancy risks within these subgroups are presented in Table 4.

**Table 4:** Pooled estimates in subgroup analysis by anticoagulation regimen

Outcome events	Warfarin throughout pregnancy, group A (n = 10)			Heparin in first trimester, then warfarin, group B (n = 5)		
	Pooled risk	Heterogeneity I^2^, %	Included studies, n	Pooled risk	Heterogeneity I^2^, %	Included studies, n
Number of pregnancies. n	375		10	100		5
Maternal outcomes						
Maternal death, %	0.91 (95% CI, 0.29-2.83)	0.00%	9	0	–	5
Total bleeding, %	3.31 (95% CI, 0.83-13.16)	84.43%	8	7.69 (95% CI, 3.67-16.14)	0%	4
Obstetric bleeding, %	2.29 (95% CI, 0.45-11.58)	72.91%	6	5.49 (95% CI, 2.29-13.20)	0%	4
Thromboembolism, %	0.01 (95% CI, 0.0-100)	88.01%	10	0.70 (95% CI, 0.02-25.16)	42.60%	5
Valve thrombosis, %	3.01 (95% CI, 1.47-6.17)	18.32%	10	6.99% (95% CI, 2.08-23.51)	27.62%	5
Pregnancy outcome						
Stillbirth, %	3.18 (95% CI, 1.22-8.33)	48.27%	8	4.84 (95% CI, 1.48-15.81)	34.42%	5
Miscarriage, %	9.95 (95% CI, 3.74-26.50)	87.92%	8	18.00 (95% CI, 11.34-28.57)	0.00%	5
Termination, %	6.41 (95% CI, 2.00-20.61)	82.65%	8	1.00 (95% CI, 0.14-7.10)	0.00%	5
Neonatal death, %	0.37 (95% CI, 0.02-7.36)	62.02%	9	0	–	5
Warfarin embryopathy, %	2.15 (95% CI, 0.72-6.43)	47.46%	9	0	–	4

*CI*, Confidence interval.

Twelve cases of valve thrombosis were observed in group A, with a pooled risk of 3.01% (95% CI, 1.47-6.17). Six of the 7 thrombotic valve events in which exact timing of the event was known (85.7%) occurred when OACs were switched to heparin after 36 weeks of gestation. Two of 12 resulted in death. In group B, valve thrombosis occurred in 8 cases with a pooled risk of 6.99% (95% CI, 2.08-23.51). Within group B, 2 women had valve thrombosis during the switch from heparin to OAC in the second trimester and 4 women had valve thrombosis after switching from OAC to heparin. Furthermore, valve thrombosis occurred in 3 women in the first trimester and 1 woman shortly before labor. Eight cases of warfarin embryopathy were described. In 5 (62.5%) of these cases, the mother took >5 mg warfarin daily throughout pregnancy.

The included studies reported a total of 11 women with mechanical prostheses who quit anticoagulation therapy during pregnancy, mostly against medical advice. Within this group, 4 (36%) stillbirths, 1 (9.1%) miscarriage, 6 (54.5%) valve thromboses, and 1 (9.1%) peripheral thrombus occurred. Four of 6 valve thromboses occurred postpartum. All women with valve thrombosis survived but had to undergo a reoperation.

One study, not included in subgroup A, reported on 31 patients who were treated with a combination of warfarin <5 mg and acetylsalicylic acid throughout pregnancy.^23^ This study reported 5 minor obstetric bleeding events due to OAC overdose, 5 peripheral embolisms, and 24 of the total 33 pregnancies in this group ended in spontaneous or therapeutic abortions.

In 15 pregnancies, LMWH (n = 13) or UFH (n = 2) was used throughout pregnancy, which resulted in a total of 14 livebirths, no maternal deaths, and 2 cases of maternal valve thrombosis during pregnancy in which surgical treatment was necessary.^15^^,^^19^^,^^23^ No detailed information on anti-Xa levels or activated partial thromboplastin time was reported.

### Oral Anticoagulant Dosage and Pregnancy Outcomes

Four studies reported pregnancy outcomes of mothers who were administered >5 mg warfarin daily during pregnancy.^12^^,^^15^^,^^17^^,^^19^ The pooled risk of adverse pregnancy outcomes was 74.24% (95% CI, 56.11-98.23), encompassing miscarriage, stillbirth, neonatal death, and warfarin embryopathy. Five studies reported pregnancy outcomes of mothers on <5 mg warfarin during pregnancy, resulting in a pooled risk of adverse pregnancy outcomes of 8.85% (95% CI, 2.70-28.99).^12^^,^^13^^,^^15^, ^16^, ^17^

### Heterogeneity

A substantial amount of heterogeneity was present within most of the outcomes in the overall meta-analysis (Table 2). Results from a univariable random-effects meta-regression model showed a significant positive association between an older age at pregnancy and risk of major bleeding during pregnancy, including all causes (*P* < .001). Year of patient inclusion was not associated with any of the clinical outcome variables. The outcomes of the meta-regression analysis are presented in Table E6.

### Quality Assessment and Sensitivity Analysis

Table E7 presents the results of the quality assessment according to the Newcastle–Ottawa Scale. All studies were of good quality, and none of them had a high risk of bias. Outcomes of the sensitivity analyses are described in the Appendix E1 and presented in Table E8.

## Discussion

This systematic review provides valuable information to improve the decision-making process concerning prosthetic valve selection in women who require MVR and are contemplating pregnancy after MVR. We presented a comprehensive overview of the reported evidence on maternal cardiac and pregnancy outcomes of women who experienced pregnancy after surgical bioprosthetic and mechanical MVR. In addition, we analyzed the maternal and pregnancy outcomes according to the practiced anticoagulation regimens, thus addressing the knowledge gap on antithrombotic management of pregnant women with prosthetic mitral valves.^26^

This study shows that pregnancy after MVR is associated with a substantially increased risk of maternal mortality, stillbirth, and neonatal death when compared with the general pregnant population.^27^ In addition, pregnancies after MVR with a biological prosthesis were associated with low risks of SVD and fetal complications compared with mechanical MVR.

### Prosthesis Type

Studies show that pregnancy after heart valve replacement with a biological prosthesis is associated with few cardiac and fetal adverse events and prophylactic anticoagulation is not necessary.^28^ However, a bioprosthesis is known for its limited durability, and implantation of a bioprosthetic valve in young patients may be associated with accelerated SVD and subsequent reoperation,^3^^,^^29^ which implies that secure timing and counseling of a pregnancy after MVR with a bioprosthesis is important to ensure a successful pregnancy without symptoms of SVD in the mother. In contrast, mechanical valves are thrombogenic and require lifelong commitment to anticoagulation therapy to prevent adverse thromboembolic events at the cost of increased bleeding risk.^4^

European and American guidelines state that the choice of prosthetic valve should be based on a shared decision-making process and in consultation with a pregnancy heart team. In addition, the European guidelines conclude that a bioprosthetic valve should be considered in women who wish to conceive and for whom MVR is unavoidable.^26^ Nevertheless, the debate of durability versus thrombogenicity remains.

Most of the pregnancies included in this meta-analysis occurred in women with a mechanical valve prosthesis (85%). Our study demonstrates increased risks of bleeding from any origin as well as pregnancy loss in women with mechanical valves compared with women with bioprostheses. Mechanical valves show a greater susceptibility to valve thrombosis, whereas none of the women with a bioprosthesis experienced this adverse event. Moreover, the observed rate of valve thrombosis is greater in the studied population as compared with nonpregnant adults receiving a mechanical valve prosthesis.^30^ In combination with the commitment to lifelong anticoagulation, our observation is in line with the outcomes of earlier studies and the advice against a mechanical MVR when contemplating pregnancy.^31^

The risk of SVD during pregnancy or within 30 days postpartum in women with a bioprosthesis in our systematic review is 3.32%. This linearized occurrence rate can hardly be interpreted, as the incidence rate of SVD increases over time, and we looked only into a period during pregnancy and 30 days’ postpartum. It is suggested that pregnancy may accelerate valve degeneration, thus leading to earlier reintervention because of hemodynamic changes and greater circulatory volumes during pregnancy. However, there is no evidence to support this theory.^32^ The included studies did not facilitate a long-term perspective of valve functioning in women with a bioprosthetic mitral valve who experienced at least one pregnancy, which makes us unable to assess the long-term durability of biological prostheses in this population. Two of the studies reported long-term durability outcomes of 87 pregnant women with a biological prosthesis in whom the occurrence of SVD was not proven to be accelerated when compared with nonpregnant patients.^10^^,^^25^ In the case of acute SVD during pregnancy, transcatheter mitral valve-in-valve replacement may be an acceptable option that ensures protection of the fetus and shows promising results for the mother.^33^ However, mid- and long-term durability is unknown, and the transcatheter procedure is accompanied with radiation, which may be teratogenous and should be taken into account.

### Anticoagulation Regimen

Our subgroup analysis of regimens A and B addresses a gap in knowledge, describing a trade-off between maternal and fetal risks regarding fetotoxicity versus maternal thrombogenicity caused by the hypercoagulable state of pregnancy and a mechanical valve in situ.^5^^,^^9^ Risks of maternal bleeding and thromboembolic events during pregnancy and within 30 days’ postpartum are present in both researched regimens. Pooled risks seem lower in regimen A. However, statistical significance was not reached, possibly due to insufficient sample sizes. Besides that, adverse maternal events in regimen B were described mainly during the transition period from warfarin to heparin or vice versa, indicating that switching is a triggering factor. In line with earlier studies, regimen A could be favorable to protect the mother.^5^, ^6^, ^7^^,^^9^ However, women on regimen A still showed increased risks during pregnancy compared with the nonpregnant mechanical MVR population.^30^

Pregnancy outcomes were similar for group A and group B. However, neonatal death and anticoagulation embryopathy risks in regimen A are 0.84% and 2.13%, respectively, whereas these events are not observed in group B. Therefore, heparin during the first trimester appears to effectively protect the fetus. Nonetheless, some argue that heparin also has negative side effects, such as thrombocytopenia, osteoporosis, or fetal malformation due to low calcium levels.^34^

High-dose administration of OACs is associated with increased fetal adverse events such as miscarriage, anticoagulation embryopathy, and neonatal death.^5^^,^^35^ Our subgroup analysis revealed that mothers on >5 mg warfarin throughout pregnancy had a 74.24% risk of experiencing an adverse fetal event compared with 8.85% if ≤5 mg warfarin was administered. These results are comparable with earlier meta-analyses.^35^

Maternal complications and pregnancy loss occurred noticeably more often in mothers who quit anticoagulants on their own behalf, which supports the thought that prophylactic anticoagulant therapy during pregnancy after mechanical MVR decreases the risk of thrombotic complications and fetal loss.

### LMWH Versus UFH

The guidelines differentiate between subcutaneous administration of LMWH or UFH during the first trimester or throughout the entire pregnancy.^9^ LMWH is believed to have more stable concentrations, resulting in superior pregnancy and maternal outcomes.^7^ We were unable to distinguish outcomes after administration of LMWH and UFH. Besides that, anti-Xa levels and activated partial thromboplastin time levels were insufficiently described. Larger and more detailed studies are necessary to draw conclusions on the maternal and fetal morbidity regarding the use of heparin during pregnancy. In the future, it could be of interest to compare patients with MVRs using OACs, LMWH, and newer-generation direct oral anticoagulants throughout pregnancy. The impact of invasive heparin administration on a mother’s quality of life during pregnancy and patient compliance should be considered when discussing anticoagulant therapy in the consultation room.

### Strengths and Limitations

We present an extensive analysis of bioprosthetic versus mechanical valve-related maternal outcomes during pregnancy after MVR. To our knowledge, this is the first systematic review assessing maternal and fetal morbidity and mortality in women conceiving after MVR. Moreover, we addressed the need for further definition of the anticoagulation regimen during pregnancy in patients with mechanical MVR. This is of clinical relevance for cardiac surgeons who choose valve prostheses, for cardiologists who advise anticoagulant regimens during pregnancy, and for obstetricians who monitor pregnancies.

This meta-analysis, however, has several limitations. First, all studies were observational and were of a retrospective nature. The inherent limitations of meta-analyses of retrospective observational studies should be taken into consideration.

It was inevitable to allow the inclusion of studies that reported on a patient populations that consisted of up to 20% of the sample size with patients who underwent a valve replacement in a nonmitral position, often the aortic position.

Specific data on the types or generations of valves implanted were insufficiently available. Therefore, comparing results between different generations of mechanical valves such as ball-in-cage versus tilting disc versus bileaflet was not possible.

In our meta-analysis, termination of a pregnancy was included as pregnancy loss, as included studies often failed to mention the exact reasons. Motivations for termination remain speculative. In the case of personal grounds, the cause of pregnancy loss was neither related to the maternal cardiac state nor to anticoagulation. Therefore, pregnancy loss because of anticoagulation may have been overestimated.

Regarding the anticoagulation regimens, we require additional information on international normalized ratios and exact dosages in order to draw a more thorough conclusion. Besides that, patient compliance to their anticoagulation regimen remains a challenging aspect, even though close monitoring was reported in most of our included studies.

Of the 15 included studies, 12 took place in developing countries. Consequently, our results should be interpreted with caution, as our study population is not fully representative for developed areas.

## Conclusions

Women with mitral valve disease who wish to conceive have an additional risk related to the childbearing and delivery, which should be accounted for when considering the valve prosthesis in young women requiring MVR. These risks need to be carefully balanced on an individual basis in an informed shared decision-making process.

**Figure 2:**
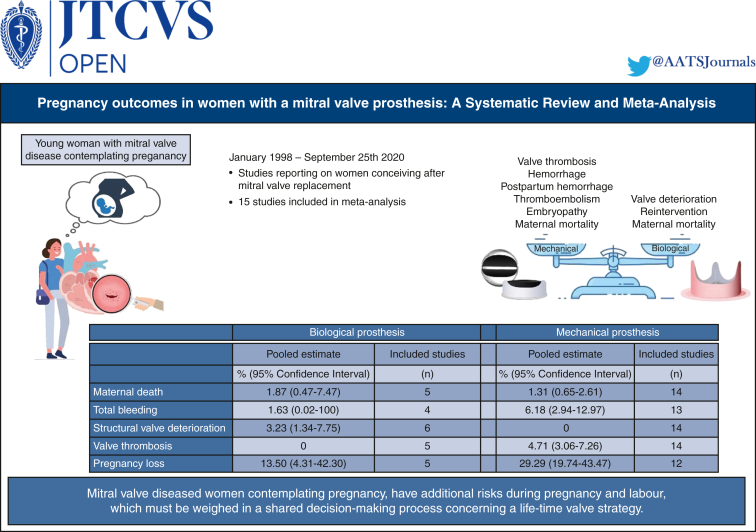
Systematic review and meta-analysis about pregnancy after mitral valve replacement.

Whereas the implantation of bioprostheses is associated with less maternal and fetal morbidity during pregnancy in comparison with mechanical prostheses, the risk of maternal mortality remains high. However, a bioprosthesis appears as the safer option during pregnancy for women who contemplate pregnancy after MVR (Figure 2).

In pregnant patients with mechanical valves, switching to heparin between weeks 6 and 12 of pregnancy increases the risks for maternal complications, whereas continuing low doses of warfarin seems to protect the mother and fetus effectively. However, regardless of which valve is implanted in the mitral position, a shared decision-making process must precede this choice. Developments of valve-in-valve surgery, MitraClips (Abbott), and optimization of bioprosthetic durability and anticoagulation therapy are key in the quality of care for this population.

### Conflict of Interest Statement

The authors reported no conflicts of interest.

The *Journal* policy requires editors and reviewers to disclose conflicts of interest and to decline handling or reviewing manuscripts for which they may have a conflict of interest. The editors and reviewers of this article have no conflicts of interest.

## Figures and Tables

**Table E1:** Data of interest for extraction: Overview of variables that were extracted from included studies

Variable	Data type
Patient characteristics	
Mean age at pregnancy	Numeric, continuous
Standard deviation of age at pregnancy	Numeric, continuous
Parity before surgery: nulliparous	Numeric, discrete
Parity before surgery: uniparous	Numeric, discrete
Parity before surgery: multiparous	Numeric, discrete
Parity before surgery: unknown	Numeric, discrete
Parity after surgery: primipara	Numeric, discrete
Parity after surgery: multipara	Numeric, discrete
Parity after surgery: unknown	Numeric, discrete
Number of patients on AC therapy	Numeric, discrete
Atrial fibrillation	Numeric, discrete
Congenital etiology	Numeric, discrete
Degenerative/calcific etiology	Numeric, discrete
Rheumatic etiology	Numeric, discrete
Endocarditis etiology	Numeric, discrete
Unknown etiology	Numeric, discrete
Procedural characteristics	
Prosthesis position: aortic	Numeric, discrete
Prosthesis position: mitral	Numeric, discrete
Prosthesis position: tricuspid	Numeric, discrete
Prosthesis position: pulmonary	Numeric, discrete
Prosthesis position: mitral + aortic	Numeric, discrete
Prosthesis position: pulmonary + aortic (ross)	Numeric, discrete
Prosthesis position: mitral + tricuspid	Numeric, discrete
Prosthesis position: unknown	Numeric, discrete
Prosthesis type: mechanical	Numeric, discrete
Prosthesis type: non-bileaflet mechanical valve	Numeric, discrete
Prosthesis type: bioprosthetic	Numeric, discrete
Prosthesis type: homograft	Numeric, discrete
Prosthesis type: autograft	Numeric, discrete
Prosthesis type: unknown	Numeric, discrete
Total number of valve prostheses implanted	Numeric, discrete

Variable	Data type
Pregnancy and maternal outcome	
Overall maternal mortality	Numeric, discrete
Cardiac maternal mortality	Numeric, discrete
Valve-related maternal mortality	Numeric, discrete
Maternal sudden unexplained death	Numeric, discrete
Pregnancy loss	Numeric, discrete
Stillbirth	Numeric, discrete
Miscarriage	Numeric, discrete
Termination: medical indication	Numeric, discrete
Termination: personal reason	Numeric, discrete
Fetal death due to preterm maternal death	Numeric, discrete
Valve reintervention	Numeric, discrete
Structural valve deterioration (SVD)	Numeric, discrete
Death due to SVD	Numeric, discrete
Reintervention due to SVD	Numeric, discrete
Nonstructural valve deterioration (NSVD)	Numeric, discrete
Death due to NSVD	Numeric, discrete
Reintervention due to NSVD	Numeric, discrete
Endocarditis	Numeric, discrete
Death due to endocarditis	Numeric, discrete
Reintervention due to endocarditis	Numeric, discrete
Thromboembolic event	Numeric, discrete
Death due to thromboembolic event	Numeric, discrete
Valve thrombosis	Numeric, discrete
Death due to valve thrombosis	Numeric, discrete
Bleeding	Numeric, discrete
Death due to bleeding	Numeric, discrete
Mortality due to endocarditis	Numeric, discrete
Thromboembolism	Numeric, discrete
Valve thrombosis	Numeric, discrete
Mortality due to valve thrombosis	Numeric, discrete
Bleeding	Numeric, discrete
Obstetric bleeding	Numeric, discrete
Mortality due to bleeding	Numeric, discrete
Cerebrovascular accident (CVA)	Numeric, discrete
Ischemic CVA	Numeric, discrete
Hemorrhagic CVA	Numeric, discrete
Transient ischemic attack (TIA)	Numeric, discrete
Ischemic TIA	Numeric, discrete
Hemorrhagic TIA	Numeric, discrete
Maternal arrhythmia	Numeric, discrete
Preeclampsia	Numeric, discrete
Pregnancy hypertension	Numeric, discrete
Heart failure	Numeric, discrete

Variable	Data type
Study characteristic	
First author	Text
Year of publication	Text
Study design	Text
Number of patients	Numeric, discrete
Inclusion period start	Numeric, discrete
Inclusion period end	Numeric, discrete
Eligibility for subgroup: AC regimen A	Binary
Eligibility for subgroup: AC regimen B	Binary
Eligibility for subgroup: Mechanical prosthesis	Binary
Eligibility for subgroup: Biological prothesis	Binary
Antithrombotic regimen	Text
Antithrombotic drug agent	Text
INR range, min	Numeric, continuous
INR range, max	Numeric, continuous

Variable	Data type
Fetal and delivery outcomes	
Vaginal birth	Numeric, discrete
Postpartum bleeding	Numeric, discrete
Cesarean delivery	Numeric, discrete
Postoperative bleeding	Numeric, discrete
Delivered alive	Numeric, discrete
Perinatal mortality	Numeric, discrete
Neonatal mortality	Numeric, discrete
Small for gestational age	Numeric, discrete
Low birth weight	Numeric, discrete
Preterm birth	Numeric, discrete
Congenital malformation	Numeric, discrete
Hydrocephalus	Numeric, discrete
Respiratory distress syndrome	Numeric, discrete
Admission to NICU	Numeric, discrete
Anticoagulant embryopathy	Numeric, discrete

*AC*, Anticoagulant; *INR*, international normalized ratio; *NICU*, neonatal intensive care unit.

**Table E2:** Anticoagulation regimens A and B

Regimen	Gestation period	Drug therapy
A	First trimester	Continue OAC
	Second and third trimester	Continue OAC
	36 wk	Switch to UFH/LMWH
B	First trimester	Switch to UFH/LMWH
	Second and third trimester	Switch from UFH/LMWH to OAC
	36 wk	Switch to UFH/LMWH

Shown is a description of anticoagulation regimens A and B. *OAC*, Oral anticoagulant; *UFH*, unfractionated heparin; *LMWH*, low-molecular weight heparin.

**Table E3:** Individual study maternal outcome estimates

Study	Maternal outcomes			
	Maternal mortality, %	Reintervention, %	Major bleeding, %	Obstetric bleeding, %
Salazar et al	3.45 (0.42-11.91)	0.00 (0.00-6.16)	–	–
Suri et al	2.94 (0.07-15.33)	–	11.76 (3.30-27.45)	11.76 (3.30-27.45)
Sadler et al	1.20 (0.03-6.53)	2.41 (0.29-8.43)	6.02 (1.98-13.50)	6.02 (1.98-13.50)
Srivastava et al	2.70 (0.07-14.16)	0.00 (0.00-9.49)	0.00 (0.00-9.49)	0.00 (0.00-9.49)
Bhutta et al	0.00 (0.00-6.98)	3.92 (0.48-13.46)	9.80 (3.26-21.41)	5.88 (1.23-16.24)
De Santo et al	0.00 (0.00-7.40)	0.00 (0.00-7.40)	–	–
Bian et al	0.00 (0.00-6.16)	–	6.90 (1.91-16.73)	6.90 (1.91-16.73)
Mazibuko et al	1.64 (0.04-8.80)	6.56 (1.82-15.95)	24.59 (14.46-37.29)	18.03 (9.36-29.98)
Samiei et al	1.89 (0.05-10.07)	–	0.00 (0.00-6.72)	0.00 (0.00-6.72)
Vijayan et al	0.00 (0.00-30.85)	–	10.00 (0.25-44.50)	10.00 (0.25-44.50)
Monteiro et al	0.00 (0.00-14.82)	–	17.39 (4.95-38.78)	0.00 (0.00-14.82)
Ayad et al	3.33 (0.41-11.53)	–	6.67 (1.85-16.20)	6.67 (1.85-16.20)
Bian et al	–	2.17 (0.06-11.53)	4.35 (0.53-14.84)	4.35 (0.53-14.84)
Iscan et al	0.00 (0.00-4.99)	4.17 (0.87-11.70)	1.39 (0.04-7.50)	1.39 (0.04-7.50)
Nelavayi et al	0.00 (0.00-12.34)	–	39.29 (21.50-59.42)	–
Pooled estimate	1.33 (0.69-2.56)	2.60 (1.28-5.29)	6.90 (3.70-12.88)	4.76 (2.64-8.59)

Study	Maternal outcomes			
	Thrombo-embolism, %	Valve thrombosis, %	Stroke, %	Heart failure, %
Salazar et al	–	–	1.72 (0.04-9.24)	–
Suri et al	2.94 (0.07-15.33)	3.85 (0.10-19.64)	–	0.00 (0.00-10.28)
Sadler et al	4.82 (1.33-11.88)	4.00 (0.49-13.71)	1.20 (0.03-6.53)	6.02 (1.98-13.50)
Srivastava et al	0.00 (0.00-9.49)	0.00 (0.00-9.49)	0.00 (0.00-9.49)	–
Bhutta et al	0.00 (0.00-6.98)	3.92 (0.48-13.46)	–	–
De Santo et al	0.00 (0.00-7.40)	4.17 (0.51-14.25)	–	–
Bian et al	0.00 (0.00-6.16)	1.72 (0.04-9.24)	0.00 (0.00-6.16)	1.72 (0.04-9.24)
Mazibuko et al	0.00 (0.00-5.87)	6.78 (1.88-16.46)	1.64 (0.04-8.80)	–
Samiei et al	1.89 (0.05-10.07)	13.21 (5.48-25.34)	–	–
Vijayan et al	–	0.00 (0.00-30.85)	–	–
Monteiro et al	0.00 (0.00-14.82)	20.00 (4.33-48.09)	–	0.00 (0.00-14.82)
Ayad et al	0.00 (0.00-5.96)	5.00 (1.04-13.92)	–	13.33 (5.94-24.59)
Bian et al	0.00 (0.00-7.71)	2.17 (0.06-11.53)	–	2.17 (0.06-11.53)
Iscan et al	8.33 (3.12-17.26)	4.17 (0.87-11.70)	–	–
Nelavayi et al	7.14 (0.88-23.50)	3.57 (0.09-18.35)	10.71 (2.27-28.23)	–
Pooled estimate	0.94 (0.22-3.96)	4.01 (2.55-6.31)	1.54 (0.46-5.14)	3.19 (1.03-9.83)

Shown in the table are pooled estimates of maternal outcomes after pregnancy with a mitral valve replacement.

**Table E4:** Individual study pregnancy and fetal outcomes

Study	Pregnancy and fetal outcomes					
	Delivered alive, %	Pregnancy loss, %	Miscarriage, %	Stillbirth, %	Termination, %	Preterm birth, %
Salazar et al	68.97 (55.46-80.46)	27.59 (16.66-40.90)	24.14 (13.87-37.17)	0.00 (0.00-6.16)	0.00 (0.00-6.16)	3.45 (0.42-11.91)
Suri et al	94.12 (80.32-99.28)	5.88 (0.72-19.68)	0.00 (0.00-10.28)	5.88 (0.72-19.68)	0.00 (0.00-10.28)	5.88 (0.72-19.68)
Sadler et al	51.81 (40.56-62.92)	43.37 (32.53-54.71)	25.30 (16.39-36.04)	4.85 (1.33-11.88)	12.05 (5.93-21.04)	14.46 (7.70-23.89)
Srivastava et al	81.08 (64.84-92.04)	18.92 (7.96-35.16)	5.41 (0.66-18.19)	0.00 (0.00-9.49)	13.51 (4.54-28.77)	2.70 (7.70-23.89)
Bhutta et al	80.39 (66.88-90.18)	19.61 (9.82-33.12)	15.69 (7.02-28.59)	3.92 (0.48-13.46)	0.00 (0.00-6.98)	3.92 (0.48-13.46)
De Santo et al	56.25 (41.18-70.52)	41.67 (27.61-56.79)	33.33 (20.40-48.41)	4.17 (0.51-14.25)	4.17 (0.51-14.25)	–
Bian et al	96.55 (88.09-99.58)	3.45 (0.42-11.91)	3.45 (0.42-11.91)	0.00 (0.00-6.16)	0.00 (0.00-6.16)	5.17 (1.08-14.38)
Mazibuko et al	67.21 (54.00-78.69)	29.51 (18.52-42.57)	19.67 (10.60-31.84)	3.28 (0.40-11.35)	6.56 (1.82-15.95)	–
Samiei et al	60.38 (46.00-73.55)	39.62 (26.45-54.00)	20.75 (10.84-34.11)	3.77 (0.46-12.98)	13.21 (5.48-25.34)	7.55 (2.09-18.21)
Vijayan et al	40.00 (12.16-73.76)	60.0 (26.24-87.84)	40.00 (12.16-73.76)	20.00 (2.52-55.61)	0.00 (0.00-30.85)	0.00 (0.00-30.85)
Monteiro et al	43.48 (23.19-65.51)	47.83 (26.82-69.41)	26.09 (10.23-48.41)	8.70 (1.07-28.04)	13.04 (2.78-33.59)	8.70 (1.07-28.04)
Ayad et al	43.33 (30.59-56.76)	56.67 (43.24-69.41)	33.33 (21.69-46.69)	23.33 (13.38-36.04)	0.00 (0.00-5.96)	6.67 (1.85-16.20)
Bian et al	–	–	–	–	–	0.00 (0.00-7.71)
Iscan et al	55.56 (43.36-67.28)	44.44 (32.72-56.64)	16.67 (8.92-27.30)	2.78 (0.34-9.68)	25.00 (15.54-36.60)	5.56 (1.53-13.62)
Nelavayi et al	67.86 (47.65-84.12)	32.14 (15.88-52.35)	0.00 (0.00-12.34)	0.00 (0.00-12.34)	32.14 (15.88-52.35)	–
Pooled estimate	66.73 (58.17-76.56)	29.08 (20.94-40.41)	15.36 (9.92-23.77)	3.49 (1.79-6.83)	3.70 (1.26-10.91)	5.56 (3.57-8.67)

Study	Pregnancy and fetal outcomes					
	Total neonatal mortality, %	Early neonatal mortality, %	Late neonatal mortality, %	Congenital malformation, %	Anticoagulation embryopathy, %	Small for gestational age, %
Salazar et al	3.45 (0.42-11.91)	3.45 (0.42-11.91)	0.00 (0.00-6.16)	0.00 (0.00-6.16)	0.00 (0.00-6.16)	0.00 (0.00-6.16)
Suri et al	0.00 (0.00-10.28)	0.00 (0.00-10.28)	0.00 (0.00-10.28)	2.94 (0.07-15.33)	–	11.76 (3.30-27.45)
Sadler et al	2.41 (0.29-8.43)	1.20 (0.03-6.53)	1.20 (0.03-6.53)	2.41 (0.29-8.43)	–	1.20 (0.03-6.53)
Srivastava et al	2.70 (7.70-23.89)	2.70 (7.70-23.89)	0.00 (0.00-9.49)	8.11 (1.70-21.91)	8.11 (1.70-21.91)	0.00 (0.00-9.49)
Bhutta et al	0.00 (0.00-6.98)	0.00 (0.00-6.98)	0.00 (0.00-6.98)	0.00 (0.00-6.98)	0.00 (0.00-6.98)	25.49 (14.33-39.63)
De Santo et al	0.00 (0.00-7.40)	0.00 (0.00-7.40)	0.00 (0.00-7.40)	2.08 (0.05-11.07)	0.00 (0.00-7.40)	–
Bian et al	0.00 (0.00-6.16)	0.00 (0.00-6.16)	0.00 (0.00-6.16)	3.45 (0.42-11.91)	0.00 (0.00-6.16)	–
Mazibuko et al	3.28 (0.40-11.35)	3.28 (0.40-11.35)	0.00 (0.00-5.87)	6.56 (1.82-15.95)	6.56 (1.82-15.95)	–
Samiei et al	0.00 (0.00-6.72)	0.00 (0.00-6.72)	0.00 (0.00-6.72)	0.00 (0.00-6.72)	0.00 (0.00-6.72)	–
Vijayan et al	0.00 (0.00-30.85)	0.00 (0.00-30.85)	0.00 (0.00-30.85)	0.00 (0.00-30.85)	0.00 (0.00-30.85)	10.00 (0.25-44.50)
Monteiro et al	8.70 (1.07-28.04)	8.70 (1.07-28.04)	–	4.35 (0.11-21.95)	4.35 (0.11-21.95)	13.04 (2.78-33.59)
Ayad et al	0.00 (0.00-5.96)	0.00 (0.00-5.96)	0.00 (0.00-5.96)	0.00 (0.00-5.96)	0.00 (0.00-5.96)	3.33 (0.41-11.53)
Bian et al	0.00 (0.00-7.71)	0.00 (0.00-7.71)	0.00 (0.00-7.71)	0.00 (0.00-7.71)	0.00 (0.00-7.71)	–
Iscan et al	2.78 (0.34-9.68)	2.78 (0.34-9.68)	0.00 (0.00-4.99)	–	–	–
Nelavayi et al	–	–	–	–	–	–
Pooled estimate	1.59 (0.88-2.86)	1.40 (0.59-3.32)	0.26 (0.02-3.18)	1.89 (0.85-4.19)	0.40 (0.03-5.41)	5.13 (1.69-15.55)

Shown are pooled estimates of pregnancy and fetal outcomes for pregnancies in women with a mitral valve replacement.

**Table E5:** Individual study characteristics

Study	Year of publication	Country	Study type	Mean year of inclusion	Mean age at pregnancy	Number of patients	Number of pregnancies
Salazar et al	1999	Mexico	Retrospective	1989	28.8	48	58
Suri et al	1999	India	Retrospective	1989	26.8	29	34
Sadler et al	2000	New Zealand	Retrospective	1982	26.4	47	83
Srivastava et al	2002	India	Retrospective	1994	27.16	30	37
Bhutta et al	2003	Pakistan	Prospective	1995	–	38	51
De Santo et al	2005	Italy	Retrospective	1989	31.4	37	48
Bian et al	2012	China	Retrospective	2007	29.4	58	58
Mazibuko et al	2012	South Africa	Retrospective	2007	24	61	61
Samiei et al	2012	Iran	Retrospective	2004	29.8	47	53
Vijayan et al	2012	Malaysia	Retrospective	2008	–	4	10
Monteiro et al	2015	Portugal	Retrospective	2007	26.3	23	23
Ayad et al	2016	Egypt	Prospective	–	–	60	60
Bian et al	2016	China	Retrospective	2011	30.5	46	46
Iscan et al	2017	Turkey	Retrospective	2003	–	57	72
Nelavayi et al	2018	India	Retrospective	2017	–	12	28
Pooled					28.0 ± 3.14	597	722

Study	Year of publication	Inclusion criteria	Exclusion criteria
Salazar et al	1999	Pregnancy after valve implantation with bovine pericardium stented prosthesis	–
Suri et al	1999	Pregnancy after valve implantation with mechanical prosthesis or biological prosthesis	–
Sadler et al	2000	Pregnancy after valve implantation at the age between 12-35 y with mechanical prosthesis, biological prosthesis or homograft	Valve prosthesis no longer available at time of study
Srivastava et al	2002	Pregnancy after valve implantation with mechanical prosthesis	–
Bhutta et al	2003	Pregnancy after cardiac surgery	Lost to follow-up, pregnancy outcome unknown
De Santo et al	2005	Pregnancy after valve implantation with mechanical prosthesis for rheumatic disease	–
Bian et al	2012	Pregnancy after heart valve replacement and use of perinatal warfarin anticoagulant therapy	–
Mazibuko et al	2012	Pregnancy after valve implantation with a mechanical prosthesis	–
Samiei et al	2012	Pregnancy after valve implantation with a mechanical prosthesis	–
Vijayan et al	2012	Pregnancy after valve implantation with mechanical prosthesis	–
Monteiro et al	2015	Pregnancy after diagnosis with valvular heart disease	
Ayad et al	2016	Pregnancy after valve implantation with mechanical prosthesis and use of anticoagulation	–
Bian et al	2016	Pregnancy after valve implantation with mechanical prosthesis while receiving low-dose oral anticoagulation therapy throughout pregnancy and terminating pregnancy by cesarean section.	Spontaneous delivery, abortion, incomplete clinical data
Iscan et al	2017	Pregnancy after valve implantation with mechanical prosthesis	–
Nelavayi et al	2018	Pregnancy in women aged <35 y after prosthetic valve implantation and use of nicoumalone during pregnancy throughout	–

Study	Year of publication	Primary outcomes
Salazar et al	1999	Valve dysfunction, mode of delivery, pregnancy outcome, embryopathy, thromboembolic event
Suri et al	1999	Deterioration of NYHA class, maternal morbidity and mortality, valve thrombosis, birth weight, Apgar score, congenital malformation, neonatal complications like intraventricular hemorrhage
Sadler et al	2000	Pregnancy outcome, preterm delivery, birth weight, structural valve failure, nonstructural dysfunction, valve thrombosis, CVA, thromboembolism, hemorrhage, endocarditis, valve replacement, cardiac failure, maternal death
Srivastava et al	2002	Pregnancy outcome, preterm delivery, mode of delivery, thromboembolic event, valve thrombosis, hemorrhage
Bhutta et al	2003	Pregnancy outcome, preterm delivery, mode of delivery, structural valve deterioration, repeat surgery, epistaxis, hematoma at episiotomy site, thromboembolism, abruptio placentae, pulmonary edema, postpartum hemorrhage, preeclampsia, arrythmias
De Santo et al	2005	Pregnancy outcome, cardiac decompensation, valve thrombosis, thromboembolism, embryopathy
Bian et al	2012	Thromboembolism, hemorrhage, echocardiography, pregnancy outcome, neonatal death, embryopathy, preterm delivery
Mazibuko et al	2012	Thromboembolism, valve thrombosis, death, pulmonary hypertension, echocardiography, pregnancy outcome, mode of delivery, embryopathy, hemorrhage, atrial fibrillation, endocarditis
Samiei et al	2012	Mode of delivery, pregnancy outcome, preterm delivery, intrauterine growth restriction, hemorrhagic complications, congenital malformation, thromboembolic complications valve thrombosis
Vijayan et al	2012	Pregnancy outcome, complications during pregnancy, postpartum complications, mode of delivery, fetal growth, fetal weight
Monteiro et al	2015	Intrauterine growth restriction, pregnancy induced hypertension, gestational diabetes, (pre-)eclampsia, hemolysis elevated liver enzymes low platelets syndrome, premature rupture of membranes, hemorrhage, premature labor, small for gestational age, pregnancy outcome, neonatal death, embryopathy, arrhythmia, heart failure worsening, endocarditis, syncope, cardiac intervention during pregnancy, thromboembolism, aortic dissection, acute coronary syndrome.
Ayad et al	2016	Hospitalization, heart failure requiring treatment, symptomatic arrhythmia, endocarditis, cardiac intervention, valve thrombosis, thromboembolic and hemorrhagic complications, acute coronary syndrome, death, intrauterine growth retardation, (pre-)eclampsia, premature rupture of membranes, premature labor, postpartum hemorrhage, placental abruption, embryopathy, pregnancy outcome
Bian et al	2016	Thromboembolism, hemorrhage, heart failure, arrhythmia, birth weight, Apgar score, neonatal death, pregnancy outcome, embryopathy, valve thrombosis, premature rupture of membranes
Iscan et al	2017	Pregnancy outcome, valve thrombosis, hemorrhage, endocarditis, preterm delivery, healthy baby
Nelavayi et al	2018	Pregnancy outcome, mode of delivery, hemorrhage, CVA, thromboembolism, valve dysfunction, birth weight, pneumonia, jaundice

*NYHA*, New York Heart Association; *CVA*, cerebrovascular accident.

**Table E6:** Meta-regression

Dependent outcome variable	Age intercept	Age coefficient, β	Year of inclusion intercept	Year of inclusion coefficient, β
Maternal mortality	−3.867	−0.000 (*P* = .99)	13.097	−0.009 (*P* = .79)
Bleeding	**5.449**	**−0.288 (*P* < .01)**	−79.117	0.038 (*P* = .056)
Thromboembolic event	1.201	−0.178 (*P* = .32)	−13.623	0.005 (*P* = .83)
Pregnancy loss	−1.401	0.008 (*P* = .91)	−10.577	0.005 (*P* = .67)
Miscarriage	−2.723	0.045 (*P* = .24)	24.762	−0.013 (*P* = .13)
Stillbirth	−1.726	−0.053 (*P* = .64)	−22.57	0.010 (*P* = .63)
Termination	−1.757	−0.020 (*P* = .81)	−68.269	0.033 (*P* = .08)
Livebirth	−0.455	0.003 (*P* = .93)	0.777	−0.001 (*P* = .93)
Maternal heart failure	3.84	−0.259 (*P* = .25)	77.819	−0.041 (*P* = .15)
Neonatal mortality	1.195	−0.175 (*P* = .22)	−36.751	0.017 (*P* = .54)

Shown are estimated intercepts and coefficients of log-transformed outcome measures in univariate meta-regression model. In meta-regression, any bleeding was significantly associated with the age at pregnancy (−0.288 [*P* < .01]).

**Table E7:** Quality assessment Newcastle–Ottawa Scale cohort studies

Study, (year)	Selection	Comparability	Outcome
Salazar et al (1999)	★★★	★★ (type of valve replacement)	★★★
Suri et al (1999)	★★★	★★ (OAC)	★★★
Sadler et al (2000)	★★★	★★ (OAC)	★★★
Srivastava et al (2002)	★★★	★	★★★
Bhutta et al (2003)	★★★★	★★ (type of valve intervention)	★★★
De Santo et al (2005)	★★★	★	★★★
Bian et al (2012)	★★★	★	★★★
Mazibuko et al (2012)	★★★	★	★★★
Samiei et al (2012)	★★★	★★ (OAC)	★★★
Vijayan et al (2012)	★★★	★	★★★
Monteiro et al (2015)	★★★★	★★ (OAC)	★★★
Ayad et al (2016)	★★★	★★	★★★
Bian et al (2016)	★★★	★	★★★
Iscan et al (2017)	★★★	★★ (OAC)	★★★
Nelavayi et al (2018)	★★★	★	★★★

Median follow-up: 9 mo + 30 d postpartum. Rationale: Due to retrospective chart reviewing, the authors were able to review all the end points that occurred during pregnancy and 30 days’ postpartum. Longer follow-up was almost never reported. Conclusion: Newcastle–Ottawa scales: good quality. Star symbols indicates rating system following the Quality assessment of the Newcastle-Ottawa Scale. *OAC*, Oral anticoagulant.

**Table E8:** Pooled maternal and pregnancy outcomes in studies published after 2010

	Studies published after 2010 (n = 9)		
	Pooled risk	Heterogeneity I^2^, %	Included studies, n
Maternal outcomes			
Maternal death, %	1.10 (95% CI, 0.41-2.92)	0.00%	8
Cardiac reintervention, %	4.47 (95% CI, 2.24-8.94)	0.00%	3
Any bleedings, %	7.31 (95% CI, 3.21-16.64)	81.81%	9
Obstetric bleedings, %	4.23 (95% CI, 1.81-9.89)	62.67%	8
Thromboembolism, %	0.80 (95% CI, 0.10-6.32)	72.80%	8
Valve thrombosis, %	4.80 (95% CI, 2.77-8.31)	24.71%	9
Stroke, %	2.05 (95% CI, 0.32-13.24)	57.22%	3
Heart failure, %	3.14 (95% CI, 0.75-13.22)	54.89%	4
Pregnancy outcome			
Pregnancy loss, %	33.36 (95% CI, 21.25-52.37)	83.67%	8
Stillbirth, %	4.22 (95% CI, 1.63-10.94)	71.26%	8
Miscarriage, %	15.98 (95% CI, 9.24-27.62)	76.00%	8
Termination of pregnancy, %	5.92 (95% CI, 1.72-19.73)	89.40%	8
Delivered alive, %	60.43 (95% CI, 49.44-73.87)	51.47%	8
Congenital malformation, %	1.60 (95% CI, 0.40-6.40)	42.32%	7
Anticoagulant embryopathy, %	0.54 (95% CI, 0.03-8.60)	68.60%	7
Total neonatal mortality, %	1.23 (95% CI, 0.30-5.10)	37.22%	8
Preterm birth, %	5.28 (95% CI, 3.28-8.49)	0.00%	7
SGA, %	6.45 (95% CI, 2.90-14.36)	0.00%	3

Shown are pooled outcomes of sensitivity analysis including only studies published after 2010. *CI*, Confidence interval; *SGA*, small for gestational age.

## Supplementary Data

Video Abstract In this video abstract the author Khargi S. explains the importance of this study. Video available at: https://www.jtcvs.org/article/S2666-2736(23)00113-4/fulltext.

## Appendix E1

### Methods|Exclusion Criteria

We excluded studies that (1) selected patients with preexisting comorbidities, (2) were not written in English, (3) reported insufficient baseline characteristics, and (4) featured study populations in which more than 20% of the total number of prosthetic valves were not implanted in the mitral position. Abstracts, posters, editorials, case reports, meta-analyses, and articles without full-text availability were also excluded from our analysis.

### Methods|Search Terms

#### embase.com

(‘heart valve replacement'/exp OR 'heart valve prosthesis'/exp OR ((implantation/de OR bioprosthesis/de) AND 'heart valve'/exp) OR 'prosthetic valve thrombosis'/de OR (((valv∗ OR tricuspid OR mitral OR aort∗ OR pericard∗) NEAR/6 (replace∗ OR substitut∗ OR prosthe∗ OR bioprosth∗ OR prothe∗ OR bioproth∗ OR artificial∗ OR implant∗ OR mechanic∗ OR metal OR biological∗)) OR (ross NEXT/1 (procedure∗ OR graft∗ OR autograft∗ OR homograft∗ OR xenograft∗))):ab,ti) AND ('pregnancy'/exp OR 'pregnant woman'/exp OR 'pregnancy outcome'/exp OR 'fetus death'/exp OR obstetrics/exp OR 'pregnancy disorder'/exp OR 'perinatal mortality'/exp OR 'maternal mortality'/exp OR 'obstetric procedure'/de OR 'obstetric delivery'/exp OR 'birth weight'/exp OR 'prematurity'/exp OR 'small for date infant'/exp OR 'neonatal intensive care unit'/exp OR (pregnan∗ OR obstetric∗ OR stillbirth∗ OR still-birth∗ OR stillborn∗ OR still-born∗ OR abortion∗ OR ((perinatal∗ OR prenatal∗ OR postnatal∗ OR peri-natal∗ OR pre-natal∗ OR post-natal∗ OR maternal∗ OR postpart∗ OR intrapart∗ OR antepart∗ OR post-part∗ OR intra-part∗ OR ante-part∗ OR fetus OR fetal OR foetus OR foetal OR neonat∗ OR newborn∗ OR gestation∗ OR baby OR babies OR maternal∗) NEAR/6 (mortalit∗ OR death OR hemmorrhag∗ OR bleeding∗ OR outcome∗)) OR obstetric∗ OR cesarean∗ OR caesarean∗ OR 'birth weight' OR birthweight OR Preterm OR Pre-term OR (premature∗ NEAR/3 (birth OR childbirth∗ OR labor OR labour OR deliver∗)) OR (small NEAR/3 (date OR gestation∗ OR age)) OR sga OR lbw OR vlbw OR elbw OR (neonatal NEAR/3 intensive NEAR/3 care) OR nicu OR nicus):ab,ti) NOT ([animals]/lim NOT [humans]/lim) NOT ([Conference Abstract]/lim) AND [english]/lim.

#### Medline ovid

(Heart Valve Prosthesis Implantation/OR Heart Valve Prosthesis/OR ((Bioprosthesis/) AND exp Heart Valves/) OR (((valv∗ OR tricuspid OR mitral OR aort∗ OR pericard∗) ADJ6 (replace∗ OR substitut∗ OR prosthe∗ OR bioprosth∗ OR prothe∗ OR bioproth∗ OR artificial∗ OR implant∗ OR mechanic∗ OR metal OR biological∗)) OR (ross ADJ (procedure∗ OR graft∗ OR autograft∗ OR homograft∗ OR xenograft∗))).ab,ti.) AND (exp Pregnancy/OR Pregnant Women/OR exp Pregnancy Outcome/OR Perinatal Death/OR Fetal Death/OR Obstetrics/OR Perinatal Mortality/OR Maternal Mortality/OR exp Delivery, Obstetric/OR exp Birth Weight/OR Infant, Premature/OR Infant, Small for Gestational Age/OR Intensive Care Units, Neonatal/OR (pregnan∗ OR obstetric∗ OR stillbirth∗ OR still-birth∗ OR stillborn∗ OR still-born∗ OR abortion∗ OR ((perinatal∗ OR prenatal∗ OR postnatal∗ OR peri-natal∗ OR pre-natal∗ OR post-natal∗ OR maternal∗ OR postpart∗ OR intrapart∗ OR antepart∗ OR post-part∗ OR intra-part∗ OR ante-part∗ OR fetus OR fetal OR foetus OR foetal OR neonat∗ OR newborn∗ OR gestation∗ OR baby OR babies OR maternal∗) ADJ6 (mortalit∗ OR death OR hemmorrhag∗ OR bleeding∗ OR outcome∗)) OR obstetric∗ OR cesarean∗ OR caesarean∗ OR birth weight OR birthweight OR Preterm OR Pre-term OR (premature∗ ADJ3 (birth OR childbirth∗ OR labor OR labour OR deliver∗)) OR (small ADJ3 (date OR gestation∗ OR age)) OR sga OR lbw OR vlbw OR elbw OR (neonatal ADJ3 intensive ADJ3 care) OR nicu OR nicus).ab,ti.) NOT (exp animals/NOT humans/) AND english.la.

#### Web of science

TS=(((((valv∗ OR tricuspid OR mitral OR aort∗ OR pericard∗) NEAR/5 (replace∗ OR substitut∗ OR prosthe∗ OR bioprosth∗ OR prothe∗ OR bioproth∗ OR artificial∗ OR implant∗ OR mechanic∗ OR metal OR biological∗)) OR (ross NEAR/1 (procedure∗ OR graft∗ OR autograft∗ OR homograft∗ OR xenograft∗)))) AND ((pregnan∗ OR obstetric∗ OR stillbirth∗ OR still-birth∗ OR stillborn∗ OR still-born∗ OR abortion∗ OR ((perinatal∗ OR prenatal∗ OR postnatal∗ OR peri-natal∗ OR pre-natal∗ OR post-natal∗ OR maternal∗ OR postpart∗ OR intrapart∗ OR antepart∗ OR post-part∗ OR intra-part∗ OR ante-part∗ OR fetus OR fetal OR foetus OR foetal OR neonat∗ OR newborn∗ OR gestation∗ OR baby OR babies OR maternal∗) NEAR/5 (mortalit∗ OR death OR hemmorrhag∗ OR bleeding∗ OR outcome∗)) OR obstetric∗ OR cesarean∗ OR caesarean∗ OR "birth weight" OR birthweight OR Preterm OR Pre-term OR (premature∗ NEAR/2 (birth OR childbirth∗ OR labor OR labour OR deliver∗)) OR (small NEAR/2 (date OR gestation∗ OR age)) OR sgaOR lbw OR vlbw OR elbw OR (neonatal NEAR/2 intensive NEAR/2 care) OR nicu OR nicus)))

#### Cochrane library

((((valv∗ OR tricuspid OR mitral OR aort∗ OR pericard∗) NEAR/6 (replace∗ OR substitut∗ OR prosthe∗ OR bioprosth∗ OR prothe∗ OR bioproth∗ OR artificial∗ OR implant∗ OR mechanic∗ OR metal OR biological∗)) OR (ross NEXT/1 (procedure∗ OR graft∗ OR autograft∗ OR homograft∗ OR xenograft∗))):ab,ti) AND ((pregnan∗ OR obstetric∗ OR stillbirth∗ OR still-birth∗ OR stillborn∗ OR still-born∗ OR abortion∗ OR ((perinatal∗ OR prenatal∗ OR postnatal∗ OR peri-natal∗ OR pre-natal∗ OR post-natal∗ OR maternal∗ OR postpart∗ OR intrapart∗ OR antepart∗ OR post-part∗ OR intra-part∗ OR ante-part∗ OR fetus OR fetal OR foetus OR foetal OR neonat∗ OR newborn∗ OR gestation∗ OR baby OR babies OR maternal∗) NEAR/6 (mortalit∗ OR death OR hemmorrhag∗ OR bleeding∗ OR outcome∗)) OR obstetric∗ OR cesarean∗ OR caesarean∗ OR 'birth weight' OR birthweight OR Preterm OR Pre-term OR (premature∗ NEAR/3 (birth OR childbirth∗ OR labor OR labour OR deliver∗)) OR (small NEAR/3 (date OR gestation∗ OR age)) OR sga OR lbw OR vlbw OR elbw OR (neonatal NEAR/3 intensive NEAR/3 care) OR nicu OR nicus):ab,ti)

### Methods|Study Selection Process

Search results were imported into the Rayyan online systematic reviews application,^E1^ after which all references published before 1998 were excluded. The remaining references were independently screened for eligibility by 2 reviewers. Disagreement on the inclusion of articles was negotiated between the 2 reviewers.

A consecutive screening on the included articles was performed to identify overlap within the populations of the included studies. In case of overlapping study populations, studies with greater data completeness or a larger population size were preferred for inclusion. In addition, studies that were excluded due to an overlapping study population within the overall study selection were once more screened for eligibility in the subgroup analyses. Finally, a cross-reference check was performed to identify studies that were missed during the literature search.

### Methods|Data Extraction Process and Definitions

The resulting datasets were cross-checked after completion by the same reviewers. In case of disagreement, a consensus was negotiated. Baseline patient , operative, and anticoagulation regimen characteristics; pregnancy outcomes; and maternal cardiac outcomes were extracted for a period up to 30 days’ postpartum. An overview of the collected variables is provided in Table E1.

Cardiac complications such as bleeding, thromboembolism, valve thrombosis, structural valve deterioration, valve-related reintervention, heart failure, and maternal mortality were defined according to the guidelines by Akins and colleagues.^E2^ Adverse maternal cardiac events were considered pregnancy-related if they occurred during pregnancy or within 30 days’ postpartum.

Fetal and neonatal mortality were defined according to the standard terminology for fetal, infant, and perinatal deaths.^E3^ Miscarriage was defined as pregnancy loss or fetal demise within the first 20 weeks of gestation, whereas stillbirth/fetal death occurred in case of prenatal fetal death after at least 20 weeks of gestation. Neonatal mortality is subdivided in early (<7 days) neonatal mortality and late (between 7 and 28 days postnatally) neonatal mortality. Pregnancy loss encompasses the total sum of miscarriages, stillbirths, and terminations of pregnancy. Preterm delivery was defined as live birth before the 37 week of gestation.

We also extracted information about prophylactic anticoagulation regimens for pregnant women with mechanical heart valves or bioprosthetic valves with chronic atrial fibrillation or previous thrombotic events in the medical history for inclusion in the subgroup analysis.

### Method|Statistical Analysis

A generalized linear mixed model was used to estimate the pooled outcome measures and corresponding confidence intervals with the transformed proportions. This method allows a pooling process with optimal estimate accuracy and minimized induced bias when studies with small sample sizes and limited numbers of event are combined, compared with traditional inversed variance methods.^E4^

### Method|Quality Assessment and Sensitivity Analysis

The Newcastle–Ottawa scale encompassed 3 quality parameters: selection, comparability, and exposure/outcome assessment.^E5^ The maximum score that could be obtained was 14, indicating a high risk of bias when the total score was 6 or lower.

The influence of potential publication bias on pooled risks was investigated by conducting sensitivity analyses by temporarily excluding the bottom quartile (by sample size) of studies in the mechanical prosthesis subgroup. The use of this subgroup for sensitivity analysis was preferred due to the heterogeneity caused by different types of valve prostheses that would be present in the overall population sample. Funnel plots could not be used to investigate publication bias as funnel plots do not allow for meaningful interpretation in case of absolute risk outcomes.^E6^

### Result|Quality Assessment and Sensitivity Analysis

Sensitivity analysis including studies published after 2010 included 9 studies reporting on 411 pregnancies in 368 patients. Pooled maternal and pregnancy risks of this analysis are presented in Table E8.

Sensitivity analyses showed that any potential publication bias did not influence our pooled risks, as pooled risks remained largely unchanged in the mechanical prosthesis subgroup after temporary exclusion of the bottom quartile of studies by sample size (before vs after exclusion: maternal mortality [1.31% vs 1.25%], obstetric bleeding [4.86% vs 4.67%], valve thrombosis [4.71% vs 4.48%], pregnancy loss [29.29% vs 30.64%], neonatal mortality [0.70% vs 0.77%]).
